# Provider Perspectives on Telepractice for Serving Families of Children Who are Deaf or Hard of Hearing

**DOI:** 10.5195/IJT.2015.6170

**Published:** 2015-07-29

**Authors:** DIANE D. BEHL, GARY KAHN

**Affiliations:** 1 NATIONAL CENTER FOR HEARING ASSESSMENT AND MANAGEMENT, UTAH STATE UNIVERSITY, LOGON, UT, USA; 2 COLORADO SCHOOL OF PUBLIC HEALTH, UNIVERSITY OF COLORADO DENVER, DENVER. CO, USA

**Keywords:** Deaf, early intervention, hard of hearing, Individuals with Disabilities Education Act (IDEA), telepractice

## Abstract

Telepractice to deliver remote Part C early intervention (EI) services to families in their home is a rapidly-growing strategy under the Individuals with Disabilities Education Act (IDEA) to meet the needs of infants and toddlers who are deaf or hard of hearing. A survey was completed within a “learning community” comprised of staff from EI programs that were implementing telepractice to learn about their specific implementation strategies and challenges they faced. Twenty-seven individuals representing 11 programs responded. The results showed great variability in hardware and software, with many raising concerns regarding security. Primary challenges reported were internet connectivity and training in skills required to deliver telepractice services. The findings from this survey were valuable in guiding future areas of investigation for the learning community and ultimately improving telepractice in the field.

Telehealth is a broad term defined by the Federal Health Resources Services Administration as “the use of electronic information and telecommunications technologies to support long-distance clinical health care, patient and professional health-related education, public health and health administration.” Within telehealth, a variety of service-specific terms have evolved. The American Speech-Language-Hearing Association uses the term “telepractice” to refer to “the application of telecommunications technology to the delivery of speech language pathology and audiology professional services at a distance” (ASHA, 2013).

Increasingly, telepractice is being used to overcome a myriad of barriers to accessing services. A telepractice session can be conducted when an in-person visit might otherwise be cancelled due to inclement weather or a minor family illness. Additionally, telepractice can allow for additional family members to join a session remotely or view a recorded session, thus encouraging family engagement. Telepractice sessions have also facilitated continuity in the receipt of services when families temporarily relocate.

Telepractice plays a particularly important role in serving families of children who are deaf or hard of hearing (D/HH) under Part C of the Individuals with Disabilities Educational Improvement Act (IDEA) of 2004. Many families face challenges accessing specialized intervention therapeutic services (Roush, 2011), especially providers with expertise in specific communication modes who are relatively few in number, such as Listening and Spoken Language and Auditory-Verbal Therapists. Families living in rural communities are often unable to access these needed therapies, and families that can access therapies may not be able to receive the optimal intensity of services. Telepractice is also being facilitated by the emergence of affordable mobile technologies such as smart phones, tablets and availability of broadband access.

In response to these challenges, the number of early intervention (EI) programs serving families of children who are D/HH via telepractice has grown substantially. Recent studies have demonstrated the effectiveness of and satisfaction with the telepractice service delivery method (Blaiser, Behl, Callow-Heusser, & White, 2013; Kelso, Olsen, Fiechtl, and Rule, 2012). Additionally, there have been many innovations in technology and connectivity, offering early intervention programs affordable options, such as use of tablets and laptops. However, there still appear to be challenges to the mainstreamed use of telepractice within the EI field. Previously reported barriers to its implementation include administrative acceptance, licensure, and reimbursement, to name a few (Brown, 2014). However, little is known regarding other aspects of telepractice implementation, such as training, equipment, and connectivity issues.

In 2005 the National Center for Hearing Assessment and Management – a federally-funded training and technical assistance center to support Early Hearing Detection and Intervention programs – brought together representatives from EI programs who are pioneers in implementing telepractice (Behl, Houston, Stredler-Brown, 2012). This learning community has served to increase networking surrounding the application of telepractice, providing a platform for participants to share successful strategies and problem solve challenges. To date, there are over 35 members who participate voluntarily in conference calls and other social media exchanges. Members represent programs throughout the country, with representation from at least twenty states.

Learning community members sought to establish a research agenda to support and improve telepractice implementation in the field. To that end, they requested a review of the current state of telepractice, characteristics of and common challenges faced by providers, hardware and software preferences, and areas of unmet need among telepractice providers serving families of children who are D/HH. In response to this request, the authors developed, executed, and now report on an online survey of EI providers serving families of children who are D/HH.

## METHODS

An online survey was developed by leaders of the telepractice learning community in fall of 2013 and administered in early 2014**.** This was a group-initiated, collaborative process with the purpose being the sharing of information among the group.

### SURVEY DEVELOPMENT

The authors developed a series of questions to ascertain specific information about the use of telepractice to serve families of children birth to three years who are D/HH. Investigators developed the initial questions with a focus on how telepractice was being implemented as well as areas of need or support desired by providers. The survey items were then sent to several members of the learning community for peer review to ensure that the questions were capturing desired information. These reviewers included a therapist using telepractice, administrators, researchers, and a doctoral student. With this feedback, questions were refined (e.g., structured responses added, deleted or modified) and additional items added (e.g., provider training and client profile). Twenty-four multiple choice items were grouped into the following seven categories:

1. Telepractice Experience/Training
2. Telepractice Clients
3. Telepractice Hardware
4. Connectivity/Videoconferencing Services
5. Technological Support
6. Telepractice Strategies/Procedures
7. Challenges in Telepractice

To capture unanticipated responses (e.g., features/functions of platform), most structured items (especially those with checklist format) included an “other” option with additional open text entry. Open-ended questions seeking comments and further details on structured items also were created for each section, enabling respondents to provide additional details about individual circumstances and important issues not captured in the structured items. The survey itself is available from the authors upon request.

The structure and length of the final survey reflected the investigators’ goal of keeping required completion time under 10 minutes to optimize the ease of completing the survey. The actual time needed to complete the survey likely depended on the extent to which respondents used the “other” category as well as the length of their open-ended responses. The survey was accessed via a link embedded in an email invitation to all learning community members.

### RESPONDENTS

The instrument was administered and data were collected via the internet using elements of the Google Drive Suite including Google Forms and Google Sheets. Targeted respondents were practitioners, e.g., speech/language therapists and deaf educators, who were actively involved in delivering telepractice to families of children diagnosed as deaf or hard of hearing (D/HH) ages birth to three years. Respondents were drawn from a convenience sample of individuals who were known to be actively conducting telepractice with this population based on their inclusion in the aforementioned telepractice learning community. An initial email request followed by an email reminder was sent out to persons on the learning community email list requesting that they complete the survey. Additionally, it was suggested that they forward the survey to other direct service providers within their program, organization, or professional community who also had experience in implementing telepractice.

### ANALYSIS

Survey data were organized using Google Sheets and Excel. Efforts were made to obtain missing responses from respondents via email and telephone follow-up. Descriptive statistics (i.e., means, percentages, number of respondents) were obtained via the automatically-calculated generation of a “report” using statistical tools in Excel and Google Sheets. Open-ended responses were analyzed through a content analysis whereby the authors reviewed, categorized, and noted frequencies of categorized responses. Open-ended comments that added to the explanation of the quantitative data were noted along with comments that complimented the quantitative closed format questions by providing novel information.

## RESULTS

A total of 27 individual responses representing telepractice providers from 11 different programs were obtained. All respondents were professionals providing direct service via telepractice (i.e., speech-language pathologists or deaf educators). Given the distribution method allowing survey dissemination by other recipients, an exact response rate could not be determined.

Programs primarily represented private/nonprofit organizations that contract with the state Part C (EI) program. The number of individuals responding from a single program varied from one to eight. One respondent was not serving the target population of families with children who are D/HH and therefore was excluded from the database.

The results are presented in the order of main survey topics described earlier. Quantitative findings are provided, followed by a summary of the open-ended responses.

### TELEPRACTICE EXPERIENCE/TRAINING

**Figure 1:**
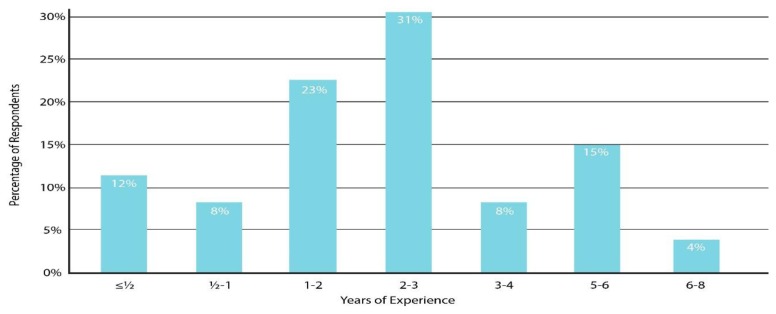
Practitioner experience in years. This figure illustrates the distribution of years of experience implementing telepractice for the survey respondents.

**Figure 2:**
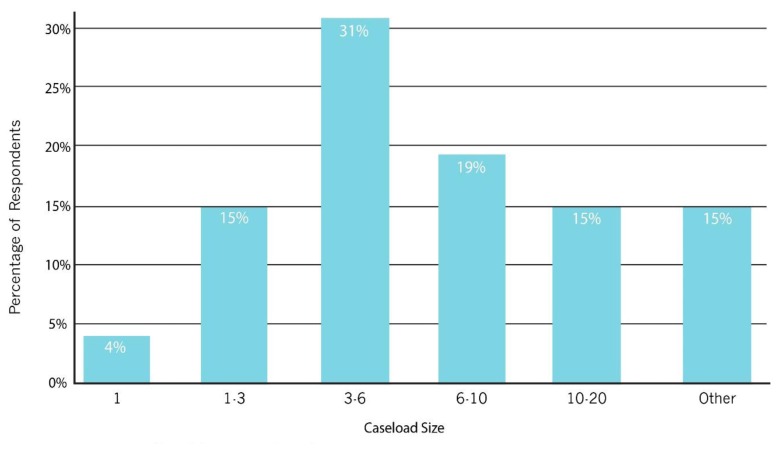
Number of clients served via telepractice. This figure summarizes the number of telepractice clients served as reported by the survey respondents.

**Figure 3:**
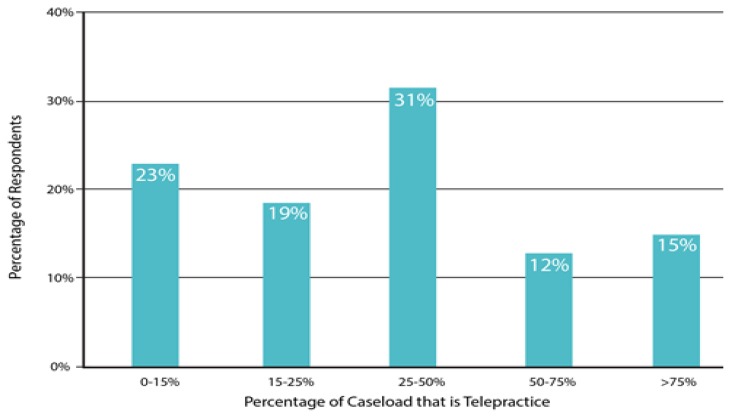
Telepractice as Percent of Total Provider Caseload This figure depicts the respondent’s reported caseload served via telepractice

As shown in Figure 1, the majority of providers had 1 to 3 years of experience implementing telepractice, with a range from less than 6 months up to 8 years. As shown in Figure 2, about 50% of the providers reported serving up to six families via telepractice. Fifteen percent (four subjects) selected the “other” option; two of these reported serving 20–30 clients, one reported serving 50–70 clients, and one reported serving 100–120 clients. In accordance with this broad range of experience, the reported cumulative number of telepractice sessions completed by each provider ranged widely, from as few as 10 by new providers to over 1300 by more experienced providers. As shown in Figure 3, most respondents reported that they do not provide services exclusively through telepractice. In fact, only 15% reported that they served three-quarters or more of their caseload via telepractice.

When asked about the training they received to do telepractice, the most commonly reported method was training received from a program or employer (46%), followed by “self-taught” (38%). Ten respondents elaborated via an open-ended query about how they went about training in telepractice. All described employing a combination of self-instruction, continuing education presentations at conferences, and networking with telepractice providers either from within their own program or via other programs. Some described learning via a “trial and error” approach obtained by jumping right into implementation or via programs that developed their own training procedures. Others described receiving training, generally in the form of workshops, from experts in the field, such as the Royal Institute for Deaf and Blind Children in Australia or experts from other universities. Comments regarding the content of the training mentioned receiving (1) training in technological aspects, such as use of equipment and trouble-shooting connection problems, as well as (2) therapeutic intervention techniques. Specific training methods used included reviewing videotapes of telepractice sessions as well as applying case studies.

### CLIENTS SERVED THROUGH TELEPRACTICE

Twenty-four of the 26 (92%) respondents reported serving families of children birth to three years, with 10 (38%) of these respondents serving this age group exclusively. Thirteen respondents (50%) also served children ages 3 to 5 years, while 11 (42%) also saw school-age children. One respondent reported providing sign language instruction to families via telepractice. Since these families typically had young children, this respondent was included in the sample. Another program reported serving mostly school-age children along with infants and toddlers.

**Figure 4:**
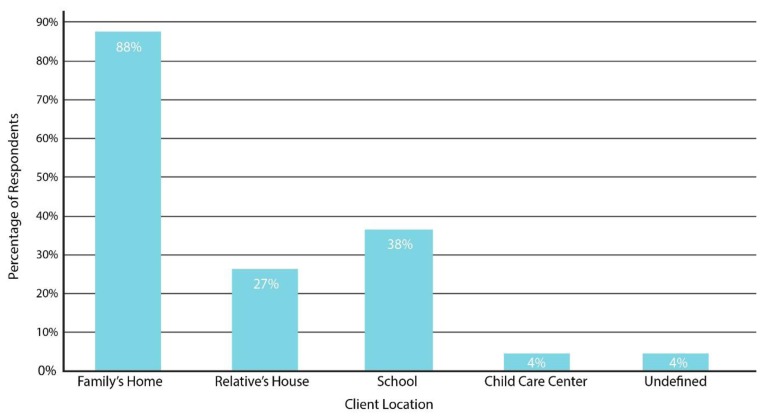
Telepractice client settings. This figure depicts the prevalence of client settings when receiving telepractice by survey respondents

Respondents were asked about the setting where their clients were located during telepractice sessions, with the opportunity to check multiple options. As shown in Figure 4, the majority (88%) reported serving families in their homes; 38% served families in a school setting; 27% served families at a relative’s home; 4% at a child care center; and 4% at an undefined international location. Respondents were asked if these clients were in a rural and/or urban area. While most (77%) reported that they serve clients in rural environment, 58% also serve families in urban locations.

### HARDWARE

As shown in Table 1, Column 1, more providers reported using Macs (54%) than PCs (38%), although several use or have used both. Sixty-two percent of providers reported using laptop computers versus 23% reporting the use of desktop computers. Interestingly, tablets (iPads) were used by 42% of providers. Almost half (46%) of respondents reported using built-in webcams, while 35% indicated use of an external webcam. A significant number of providers (35%) reported using external speakers. Only 11% reported that they used a headset with a microphone.

Two providers commented via open-ended responses that they didn’t know what hardware all of their clients were using, but all did respond. As shown in Table 1, Column 2, providers reported that more clients were using PCs (46%) than Macs (23%). Approximately 58% of providers reported that clients had used a tablet (all iPads, no Android-based tablets) for telepractice sessions, while 54% have clients with laptops and 46% have clients with desktop computers. Note that these categories are not mutually exclusive. The majority (85%) of providers reported that clients had the ability to move their devices to different locations within the home during a session. In regard to additional hardware used by clients, 38% of providers reported that their clients had used an external webcam and 35% had used external speakers.

About 65% of respondents indicated that they had loaned hardware to client families. From the open-ended comments, this was mostly for peripheral devices (external webcams and echo-cancelling speakerphones) to upgrade the sound or picture. Although laptops and desktops were occasionally loaned out, especially for research studies, the primary computing device reportedly loaned to families was an iPad or iPad mini; this was the hardware of choice for providers from one program that sought to use a standardized platform. Free-response comments about hardware reinforced the closed format questions. In general, providers and families used an array of hardware depending on availability.

Respondents also elaborated via open-ended responses on their support to families in need of equipment or enhanced internet connectivity. Programs often loaned equipment to families, with some programs creating “loaner equipment libraries.” In fact, one respondent explained that their goal is for all of their clients to use loaned equipment.

### CONNECTIVITY/VIDEOCONFERENCING SERVICES

Respondents reported little diversity in regard to the videoconferencing platforms they have used and their current primary videoconferencing service. As shown in Table 2, the most commonly used videoconferencing service was FaceTime (50%), which is a proprietary Apple product requiring the use of Macs, iPads, iPods, or iPhones. Nearly half (46%) of respondents reported using Skype, but only 15% used it most frequently. A small number had tried other platforms such as Vidyo, Zoom, GoToMeeting, WebEx, and Adobe Connect. Fifteen percent of respondents reported using solely a proprietary system specific to their program, and 8% reported using a propriety system equally as often as Jabber. The extent to which families received financial support from programs to cover internet connectivity also was investigated. Twenty-three percent indicated they pay for or subsidize connectivity charges. One respondent indicated that connectivity charges, as well as hardware, were provided to families by a third-party entity.

When asked how satisfied they were with the quality of the videoconferencing experience, only 15% were completely satisfied and 4% completely dissatisfied based on a 5-point scale (1=Totally Satisfied, 5=Totally Dissatisfied). Skype received the lowest rating (median =3), while the other systems had a median rating of 2.

Respondents then reported specific areas in which they experienced problems. Problems with internet connections were reported by 88% of respondents; “video” and “audio” were specific problem areas cited by 62% of respondents. Twenty-three percent of respondents reported problems with their clients’ hardware while only 8% of providers had problems with their own hardware.

Providers’ open-ended responses highlighted the videoconferencing challenges faced during telepractice sessions. Many highlighted problems attributed to poor bandwidth on the client end, especially when using Skype. This was reported to result in a pixilated picture and freezing of the video and audio. Additionally, one respondent reported that when a child or something in the environment makes enough noise in the background, it can limit the ability of the provider to hear the targeted child and/or family member. This often interferes with the flow of the sessions and can become frustrating. One respondent reported experiencing disruptions of sign language motions due to poor video quality. Problems were reported as less frequent among those who used FaceTime. Some providers reported that they had been satisfied with the technology support provided by software platforms such as Vidyo, which requires a paid subscription.

To obtain objective information about the bandwidth available, respondents were asked to conduct a speed test while they were filling out the questionnaire. A link (http://www.speedtest.net) was inserted into the questionnaire and respondents were asked to fill in the download and upload speeds that came back on a report screen, along with their ISP (internet service provider). The reported speed test results varied greatly from a down/up speed high of 115/127 Mbps to a low of 1.0/0.74 Mbps with a mean of 42/24 Mbps and a median of 25/9 Mbps.

### TECHNOLOGY SUPPORT

Three questions were asked to determine the types and adequacy of technology support services to assist the providers when problems arose. When asked about the availability of tech support using a scale of 1–5, with 1=always available and 5=never available, only 23% reported that such support was always available, with a median rating of 3. When asked to rate the adequacy of this tech support (1=Problem always solved, 5=Problem never solved), respondents reported a median rating of 3. Only 23% reported their problem always was solved. Respondents were asked to specify how they accessed technical support. Interestingly, 38% had support onsite and 50% indicated they had on-call support, either by phone or online. Several indicated they had multiple sources of support, while 15% indicated they provided all technical support themselves. To assess the degree to which respondents tried to minimize the need for third-party tech support, we asked about their own training in how to troubleshoot. Approximately 42% of providers indicated they received troubleshooting training, and 27% indicated their clients also received such training.

### TELEPRACTICE STRATEGIES/PROCEDURES

**Figure 5:**
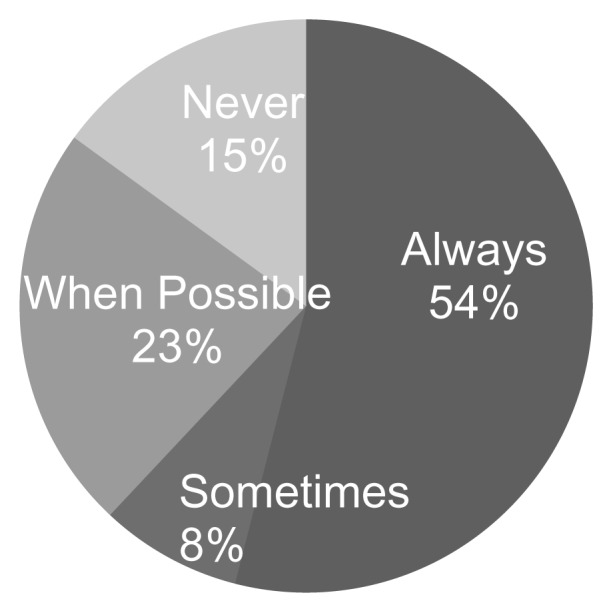
Responses to “How often do you conduct an in-person meeting before telepractice service delivery?”

In response to “How often do you conduct an in-person visit prior to telepractice service delivery,” 54% of respondents indicated they always first meet clients in person before starting telepractice, 8% said sometimes, 15% responded that they never meet clients in person before starting telepractice, and 23% responded “whenever possible” (see Figure 5). Answers to free response items reflected an overall perspective that the necessity of an in-person meeting prior to beginning telepractice depends on the family circumstances and practicalities. Some providers stated that seeing the family in-person is optimal in that it provides an opportunity to establish rapport, to observe the family’s culture and home environment, and to learn important information about the child’s audiological needs. Several commented that an in-person evaluation typically occurs prior to the delivery of services, providing an opportunity for the family to meet with the provider prior to starting telepractice

In general, providers conveyed that an in-person meeting is dependent on (1) the provider’s belief that an in-person visit is important for establishing rapport, (2) the family’s comfort level with using technology, and (3) whether a provider is only accessible via telepractice, such as when the family lives many hours away. In fact, one provider expressed that “arbitrary requirements (i.e., a requisite initial in-person meeting) will not best meet the needs of all clients and may significantly limit access for some clients who could otherwise benefit from the use of telehealth technologies.” Providers serving preschool or school-age children reported that they make sure to hold videoconferences with the child’s teacher and telephone the child’s family prior to beginning telepractice services. Some respondents described other preparation procedures prior to starting to deliver telepractice services. One responded stated, “Before transitioning to online services, I ask the parent to bring materials to an in-person visit at the clinic to ensure the caregiver is comfortable selecting developmentally appropriate materials and to evaluate their willingness to follow through with the strategies learned in therapy.” Additionally, one provider reported that “some Part C funders will not allow billing when the services are not in-person at least a portion of the time,” reflecting the importance of adhering to state government regulations.

When asked in an open-ended question to describe their telepractice process, respondents indicated that the telepractice session itself is reported as being based on the Individualized Family Service Plan (IFSP) or the child’s Individualized Education Plan (IEP). Providers in EI programs described their sessions as being focused on coaching the caregiver to take the lead in conducting the activities, providing feedback to the caregivers. In addition to the live videoconferencing that occurs during a telepractice session, respondents were asked about other technology-assisted tools or processes they used during their sessions. As shown in Table 3, 77% of respondents reported that they share electronic documents with clients after sessions, such as follow-up activities or session summaries. A slightly smaller number of respondents (65%) share electronic documents with families prior to the session. Twelve to 23% of respondents respectively reported that they are interested in employing these strategies although they do not currently do so. Half of respondents currently record sessions for their own review while only 23% share recordings with clients. Of the respondents who did not report recording sessions, 31% indicated they would like to do so for their own purposes and 35% indicated they would like to have recordings for their clients to view.

Answers to open-ended questions provided insights into the type of preparation and follow-up activities that occur in relation to telepractice. Comments reflected the importance of preparation activities for many providers. These reported preparation activities include: (1) confirmation of the session time and date, (2) sending lesson plans or written documents reflecting the goals of the session, and (3) identifying the toys, food and other materials needed for the upcoming lesson. Some providers stated that they mailed needed toys and supplies to families several days ahead of time, particularly when families were not able to afford toys or other materials. One provider described providing these “asynchronous” services when serving families that live in vastly different time zones. In such cases, families sent video recordings of their interactions with their child and the provider provided feedback after viewing. Providers serving school-age children reported that they planned sessions in coordination with the classroom curriculum, and classroom topics or events were incorporated into telepractice sessions.

Communication following a telepractice session was reported to typically occur via email, with providers sending reports to families that describe what occurred during the session, how these activities link to the child’s IFSP goals, and ways to incorporate therapeutic activities into daily routines to continue to progress the child’s skills. Emails and telephone interactions also reported as occurring between sessions. One provider reported that the amount of paperwork and documentation required for each individual family can be overwhelming to her, especially if the family is seen mostly via telepractice.

#### PERCEIVED CHALLENGES IN IMPLEMENTING TELEPRACTICE

Providers responded to an open-ended question inquiring about how their use of telepractice could be improved. Some providers reported a need for improvements related to equipment, such as funds for more loaner equipment for families, as well as special equipment for recording purposes. A storage system for video recordings also was identified as a need, along with user-friendly mechanisms (websites/libraries) to share information with families outside of visits. Other needs pertained to training and enhancing ways to share information. Some identified the need to better prepare families for telepractice by offering inservice training to help them understand the process. Software platforms to allow for multi-party videoconferencing were mentioned, along with screen sharing capacity to enhance lessons and review reports. Another respondent described a desire to create an “online home” for accessing resources as well as chat rooms, etc.

## DISCUSSION

This survey reported on telepractice implemented by EI providers serving families of young children who are deaf or hard of hearing (D/HH). The survey was an activity originated by members of a telepractice learning community that serve this population to better understand the delivery mechanisms and needs surrounding telepractice. The findings shed light on the types of equipment and software being used, the extent to which specific training has been obtained, as well as the perceived benefits and challenges of providing services via telepractice. This information was sought to foster improvements in the telepractice implementation by guiding future interactions of the learning community members. As a result, the convenience sample and the narrow population surveyed (telepractice providers serving infants and toddlers who are D/HH) limits the generalizability of the findings. In spite of this limitation, the results still provide a snapshot into current telepractice procedures employed in the field of Part C EI.

Both providers and families likely used different types of hardware during the course of telepractice. They may have used iPads exclusively or just when at a temporary location (relative’s house or on vacation). Instead of expensive systems used in the past, such as Polycom, Lifesize, and Tandberg room-based videoconferencing hardware systems and dedicated lines, the majority of programs are now using laptops and tablets along with free or low-cost web-based (cloud-based) videoconferencing services and voice-over-internet protocols (VoIP). Telepractice software and hardware technology has changed from requiring significant infrastructure to becoming a method that is now part of mainstream social media technologies. For example, headsets were often required in past years to reduce echoing, yet their infrequent use reported by these providers likely reflects the greatly improved echo canceling algorithms in newer computers and tablets. Providers appear to be getting away from desktop systems and moving to laptops and tablets (particularly iPads) due to their affordability, portability, and ease of use. For example, one provider commented that the iPad allowed the caregiver to position the camera at an angle such that the provider could better view the child’s responses when engaged in a therapeutic activity. As a result of these rapid changes in hardware and software platforms, the cost-effectiveness of telepractice will likely increase in relation to the growing cost of travel and provider hours required for traditional in-person visits. This potential cost savings may be persuasive to hesitant administrators (Cason, Behl, & Ringwalt, 2013), provided that compliance with HIPAA is maintained.

Results from this survey suggest that unresolved technical problems are a common experience for most practitioners in this sample, and that obtaining technical support is a challenge for many. Given the constantly-evolving technologies that appear to be making telepractice more affordable, flexible, and reliable, this need for technical support may decrease. The confidence of users and administrators can be enhanced by using strategies that increase the likelihood of a trouble-free videoconferencing session, such as providing loaned equipment that can be programmed or “locked down” to reduce the risk of overloading the computer with viruses or downloads.

Privacy and security are factors that remain of critical importance when conducting telepractice. Although this survey did not query specifically about these issues, it is a topic relevant for training, especially when providers reported using platforms that have inherent risks to security and privacy, such as Skype and FaceTime. These findings point to the need for training specifically related to adherence to privacy and security requirements.

In terms of perceived challenges in delivering telepractice, connectivity is still a major problem. Universities and other institutional settings often have internet speeds significantly above what is necessary for good quality videoconferencing. Vidyo, for example, recommends minimum download/upload speeds of approximately 1.5 /1.5 Mbps for HD service, while FaceTime states that it requires a minimum of 1.0/1.0 Mbps for HD. However, these recommended download and upload speeds are needed for operating these programs alone; other programs running concurrently result in fluctuation and decreased quality.

There are still many rural areas that do not have access to high-speed internet, and those families that do have access are frequently challenged by the cost of the service (FCC, 2011). Connectivity in urban areas also can be a challenge as the growing demand for continuous service fills the existing capacity of the internet “highway.” It is important to point out that the internet speed test used in the survey is merely a cross-sectional “spot check”; speeds can fluctuate markedly depending on time of day and number of users sharing the connection. This fluctuation is often a major challenge faced by practitioners trying to serve families, often during periods of high broadband demand. This, too, is a pertinent topic for the learning community to explore in terms of connectivity options.

Fortunately, in many areas such as urban centers, available bandwidth may soon surpass the requirements for the consistent HD quality videoconferencing desirable for telepractice. This will be driven, in part, by robust competition among videoconferencing providers with improved compression algorithms that have entered the market in the last few years, as well as from the bandwidth requirements of ultra-high definition displays (TVs) just coming onto the market. Until then, and for a while in many rural and remote areas, practitioners will depend upon the ongoing improvements in technology being made available by the competition among videoconferencing services. One strategy that may be useful in the interim is to purchase upgraded service (higher bandwidth) on the client side via their internet service provider. Some programs in the survey reported having to subsidize lower-income families in these situations.

In keeping with its original intent, this survey provides a snapshot of how telepractice fits in the overall provision of services to children who are D/HH and their families. The finding that telepractice is often provided in tandem with in-person home visits suggests that telepractice is not strictly viewed as taking the place of in-person visits. Rather, it is a useful strategy in enhancing service delivery. Again, telepractice is being used by most providers as a way to increase access to services for families, providing access during times of bad weather, to avoid exposure to illness, or to simply allow providers to spend their time providing services rather than spending hours on the road. Thus, it should be viewed as one more valuable approach to meeting the needs of children and families.

This survey also points to areas for which further exploration is needed. For example, the survey reflects a need for systematic training for providers to prepare them for implementing telepractice. None of the respondents in our sample reported that they had received telepractice training in a university setting as part of pre-service education.

Based on these results, providers have primarily learned to implement telepractice through personal experience, training offered by their employer in their program, from workshops at professional meetings, as well as via learning from other programs who have been implementing it. The field may benefit from training curricula as well as more systematic and comprehensive “coaching the coaches” models. This is a likely direction for the future, and it will become increasingly important as the prevalence of telepractice grows. Additionally, training in the use of other electronic methods to enhance telepractice, such as screen sharing, facilitating electronic exchange of documents and video recordings, would support provider interest in these tools.

As mentioned previously, there are limitations that are important to note. First, the number of respondents is relatively small, although it is representative of the relatively few programs serving children who are D/HH through telepractice. Replication of this survey with a larger population of providers serving the broader population of children with special needs would provide an opportunity to learn more about the generalizability of these findings as well as to learn to what extent some solutions, such as more systematic training for providers, have been addressed. Additionally, it is important to note that a significant number of providers responding to this survey were all employed by one program resulting in a possible over-representation of this hardware and software. Again, replication of this survey would reinforce these findings.

In spite of these limitations, the results of this survey do inform the field of EI in regard to applying telepractice to meet the needs of children and families. The results reflect the provider opinion that telepractice is perceived as a valuable tool, but more importantly, it points to the technological advancements as well as training that are needed to ensure telepractice is implemented in an effective manner. These findings can also serve as impetus for continued research and dialogue about this ever-growing practice. The information gleaned from this survey offers much to guide the direction and shared learning activities of the telepractice learning community, with the intention of improving the quality of telepractice in serving children who are D/HH and their families. In turn, these findings inform the broader telehealth field about the existence of this application of telepractice and its importance in supporting the Part C EI system. Together, the value of telehealth can be brought to the forefront through its use by allied health professionals, within the home setting, and with diverse clients.

## Figures and Tables

**Table 1:** Providers and clients reported (by providers) to use each form of hardware

	Providers	Clients
*Platform:*		
Windows	38%	46%
Mac	54%	23%
Form Factor		
Desktop	23%	46%
Laptop	62%	54%
Tablet		
— iPad	42%	58%
— Android	0%	0%
*Built-in and Peripheral Hardware:*		
Built-in Webcam	46%	54%
External Webcam	35%	38%
Pan-Tilt-Zoom Camera	4%	4%
Document Camera	12%	0%
Echo-cancelling Speakerphone	15%	12%
External Speakers	33%	33%
External Monitor	19%	15%
External Mic	19%	30%
Headset w/Mic	11%	4%

**Table 2:** Videoconference Services Used for Telepractice

Platform:	Which videoconferencing service(s) do you use? (all)	Which Videoconferencing services do you use the most?
FaceTime	65%	50%
Skype	46%	15%
Proprietary	15%	15%
Proprietary/Jabber	8%	8%
GoToMeeting	12%	0%
Vidyo	8%	4%
Zoom	4%	0%
VSee	4%	0%
Adobe Connect	4%	0%
WebEx	8%	0%
Tandberg	4%	0%
Google+/Hangouts	4%	4%
Missing	4%	4%

**Table 3:** Practitioner’s Telepractice Process

Component	Currently Use	Would Like to Use (Not Currently Using)
Post-session docs (todo’s, checklists)	77%	12%
Pre-session docs (instructions, plans, books)	65%	23%
Video record sessions for practitioner review	50%	31%
Video record sessions for caregiver review	23%	35%
Electronic patient record or session notes	19%	27%
Digital whiteboard	19%	27%
Desktop sharing	15%	15%
Other	15%	-
Scheduling system	8%	19%
None of the above	4%	4%
